# A process evaluation of user fees abolition for pregnant women and children under five years in two districts in Niger (West Africa)

**DOI:** 10.1186/1472-6963-9-89

**Published:** 2009-06-03

**Authors:** Valéry Ridde, Aissa Diarra

**Affiliations:** 1 Department of Preventive and Social Medicine, Faculty of Medicine, University of Montréal, 3875 Saint-Urbain St, Montréal, QC, Canada; 2 Research Centre of the University of Montreal Hospital Centre, CRCHUM, Montréal, QC, Canada; 3 Laboratoire d'Études et de Recherches sur les Dynamiques Sociales et le Développement Local, LASDEL, Niger

## Abstract

**Background:**

African policy-makers are increasingly considering abolishing user fees as a solution to improve access to health care systems. There is little evidence on this subject in West Africa, and particularly in countries that have organized their healthcare system on the basis of the Bamako Initiative. This article presents a process evaluation of an NGO intervention to abolish user fees in Niger for children under five years and pregnant women.

**Methods:**

The intervention was launched in 2006 in two health districts and 43 health centres. The intervention consisted of abolishing user fees and improving the quality of services (drugs, ambulance, etc.). We carried out a process evaluation in April 2007 using qualitative and quantitative data. Three data collection methods were used: i) individual in-depth interviews (n = 85) and focus groups (n = 8); ii) participant observation in 12 health centres; and iii) self-administered structured questionnaires (n = 51 health staff).

**Results:**

The population favoured abolition; health officials and local decision-makers were in favour, but they worried about its sustainability. Among health workers, opposition to providing free services was more widespread. The strengths of the process were: a top-down phase of information and raising community awareness; appropriate incentive measures; a good drug supply system; and the organization of a medical evacuation system. The major weaknesses of the process were: the perverse effects of incentive bonuses; the lack of community-based management committees' involvement in the management; the creation of a system running in parallel with the BI system; the lack of action to support the service offer; and the poor coordination of the availability of free services at different levels of the health pyramid. Some unintended outcomes are also documented.

**Conclusion:**

The linkages between systems in which some patients pay (Bamako Initiative) and some do not should be carefully considered and organized in accordance with the local reality. For the poorest patients to really benefit, it is essential that, at the same time, the quality of services be improved and mechanisms be put in place to prevent abuses. Much remains to be done to generate knowledge on the processes for abolishing fees in West Africa.

## Background

For Africa to attain the Millenium Development Goals, the challenges remain vast, especially with regard to infant and maternal mortality [1]. Improved access to health care contributes to achieving these objectives [2,3]. However, in many countries this access is very much constrained because patients must pay for services, whereas abolishing user fees has the potential to save around 230000 children under age five each year in 20 African countries [4]. "Alleviating financial barriers must become a priority for policymakers if their will is really to accelerate the reduction of maternal and perinatal mortality in the developing word," a group of researchers recently affirmed [3]. Our study was carried out in Niger (West Africa), where, in 1995, the government decided to generalize the Bamako Initiative (BI), user fees, community participation, and the utilization of essential drugs. The objective was to improve the quality and utilization of health care services. Ten years later, service utilization remains very low, especially because the State has reduced its financial contribution and not all patients have the means to pay [5,6]. In Africa, user fees have constrained access to care [7,8], notably in Niger [6,9], pushing some families into poverty [10]. Alternative strategies against health care exclusion that are based on community-based health insurance (CBHI), on family solidarity, or even on targeted exemptions have not yet produced the desired results. There is no evidence of the progressiveness of CBHI, they are not targeted to the worst-off, and their penetration rate is low (maximum 10%). Family solidarity is eroded, and exemption schemes either do not work or have been co-opted by other than the needy [11-15]. In fact, implementing targeted exemptions in Africa is a challenge [16].

### User fees abolition

Decision-makers are searching for solutions, one of which is the abolition of user fees for all, or at least for easily targeted beneficiaries (e.g. children under five and pregnant women). Except for some recent studies in Ghana and Senegal, where user fees for deliveries were abolished [17-19], there is, as of yet, no evidence on this subject in West Africa, and particularly in countries that have organized their health care system on the basis of the Bamako Initiative (BI). However, some southern and eastern African countries have abolished user fees. In most cases, results have been immediate and positive, with increased utilization of health care services among children under five. In Uganda, new cases treated increased by 18.5% for children under five nationwide after the abolition in 2001 [20], and 27% in another study in 10 districts [21]. The poor had benefited more than others in terms of health care utilization [20,22,23] but not for catastrophic health expenditures [23]. By way of contrast, in Ghana the richest households had a decline in out-of-pocket payments of 21%, while the decline in poor households was 13.18%. However, the proportion of facility-based deliveries increased in every socio-economic quintile, but the greatest increases were in the two poorest [24]. In South Africa the positive effect of abolition on curative care for children in 1994 (+77%) was undermined by little or no increase in preventive care (immunization and growth monitoring) [25]. In Zambia, utilization of rural health facilities was estimated to have increased 55% over the 12 months following the removal of user fees in 2006 (excluding children under five) [26]. In Burundi, three months after fees were abolished at hospitals in May 2006, average monthly outpatient visits by children had increased 42% and inpatient stays, 87% [27]. In Madagascar, the (temporary) abolition of user fees in 2002 doubled the average monthly visits in December compared with the previous year (117 vs. 208), and health staff perceived the main reason to be the abolition of user fees. Researchers affirm that abolition of user fees is associated with a 22% increase in visits to health centres, after controlling for supply effects (i.e., proxy of quality) [28].

As we see, researchers have concentrated primarily on demonstrating the outcomes of this abolition policy. Our literature review shows that documenting the process has not received much attention from researchers [29]. There were, however, exceptions, and these experiences have shown the importance of the implementation process and the need for careful action [30]. To implement the abolition and to support the subsequent increase in utilization requires sufficient increases in funding [20,21]. Otherwise, repeated problems with drug stocks could have a negative effect on utilization [31], as in Zambia, and on staff morale, as in South Africa [32]. In Uganda, the disappointing failure of abolition to protect the poor against poverty could be explained by the unavailability of drugs in health centres [23]. Leadership and active communication strategies are also needed. In Madagascar, political uncertainty and imperfect communication about the abolition were partly responsible for the implementation gap in rural communes [28]. Front-line health workers and the population must be informed and work together at the beginning of the implementation phase. If not, there is a risk of disillusionment or obstruction on the part of health staff, as was the case in South Africa [32,33], or even a tendency to compensate (through informal payment or private practice) for revenue lost [21]. The issue of provider incentive must be taken seriously [34].

Given the current state of knowledge, abolishing user fees is one of the measures recommended by scholars to decision-makers to redress the inequity of access to health care [2,35]. The final report of the Health Systems Knowledge Network of the WHO Commission on the Social Determinants of Health clearly advised to "remove user fees for public services" [2]. Aid donors like DFID (United Kingdom) [1], the Humanitarian Aid Department of the European Commission [36], and the World Bank [37] now also favour supporting countries who want to abolish user fees. WHO Director-General Margaret Chan said, "if you want to reduce poverty, it makes sense to help governments abolish user fees" [38]. However, information on how to implement this abolition remains scarce. To support decision-makers in West Africa, especially those who have been implementing the Bamako Initiative since 1987, this paper presents the process evaluation of the first intervention done in two districts (one million inhabitants) in Niger in 2006–2007. Lessons drawn from this analysis may be useful to decision-makers in other West African countries as well as in Niger, whose government decided to extend this policy nationwide as of April 2007.

### Intervention Background

Niger is a West African country of 14 million inhabitants. The United Nations estimated that Niger is "not on track" to reach Millennium Development Goals 4 and 5 (under-five mortality rate; maternal mortality) by 2015. The 2015 target for under-five mortality rate is 107 per 1000, and the latest figure in 2005 was 253 [39]. Niger ranks third highest (out of 46) in Africa in terms of the probability of dying under five years of age (WHO, ). The maternal mortality ratio of 1.800 per 100 000 live births in 2005 is the second highest in Africa after Sierra Leone. Concerning poverty, the UN estimated that, in 1995, 61% of the population had less than one dollar per day in purchasing power parity (PPP 1993). Forty percent of children under five are underweight, just 33% of children under five with fever receive antimalarial drugs, and only 7% sleep under an insecticide-treated net. Among women, 18% of live births in 2006 were attended by skilled health personnel, the third lowest rate in Africa after Chad and Ethiopia. The contraceptive prevalence is 11% (WHO, ) and 46% of women aged 15–49 were attended at least once by a skilled health provider during pregnancy in 2006 [39]. Only 4% of mothers in the poorest quintile used skilled care at delivery, compared with 63% in the richest quintile [3]. In 2006, according to WHO, 4% of the gross domestic product is devoted to health expenditure, 53% by the government and 47% private. Private expenditure on health is 85% out-of-pocket, i.e., user fees. Per capita total expenditure on health is $27 (international dollar rate). In 2005, the government decided to make caesareans free (implementation started in 2006), but aside from certain specific pathologies (e.g. tuberculosis, leprosy), all other services were not.

In 2005, Niger underwent a major food crisis. On this occasion, the German non- governmental organization HELP (Hilfe zur Selbsthilfe e.V.) intervened. The staff observed that, despite food being distributed, malnutrition was still a problem. The NGO decided something had to be done about health care access, which is one of the determinants of malnutrition, according to the UNICEF [40] framework. It was then that the Humanitarian Aid Department (ECHO) of the European Commission asked the NGO to organize an intervention aimed at abolishing user fees in two districts to increase service utilization among children under five (curative care and medical evacuation) and pregnant women (prenatal care, deliveries, and medical evacuation). Each of these districts (Mayahi and Tera, 1 000 km apart) has approximately 500000 inhabitants, a district hospital, and around 20 health centres (Table 1).

**Table 1:** Comparative indicators for the two districts

Indicator	Mayahi	Tera
Number of Type 1 integrated health centres (CSI) (2005)	20	23

Management of cost recovery system^(a)^	Independent	Single

Total number of inhabitants (2005)	464044	475557

Rate of utilization, curative services (2005)	Non Available	0.13

Percentage of babies with low birth weight (2005)	Non Available	8

Rate of BCG vaccination (2005)	94	89

Proportion of CSIs with cost-recovery schemes in 2000	96	100

Name of the region^(b)^	Maradi	Tillaberi

Proportion of inhabitants living in poverty (2005)	79.7	68.9

Proportion of the poor without schooling (2005)	60.7	71.7

Proportion of households living more than 60 minutes away from a CSI or health post (2005)	45.1	45.5

Majority group	Haoussa	Songhaï

Rate of vulnerability to food insecurity (2004)	minimal	minimal

Gross rate of schooling 1999–2000	30.7	28.9

Source: SNIS 2005, Rapport sur le développement humain 2004, Document de lutte contre la pauvreté 2006

^**(a) **^Independent fund = each CSI collects and manages the user fees, which are retained by the CSI; Single fund = each CSI collects user fees, which are then centralized at the district level and managed by a community manager for all the district's CSIs.

^**(b) **^The above data are based on regions, since almost no database in Niger provides district-level data.

A health system in a district in Niger is organized at three levels. Health posts (where payment for services continues at the time of the study) serve the population of one village and provide very basic outpatient curative and preventive care. They are managed by a community-based health worker, who is trained for three months and paid by the government. Most health posts function poorly and are rarely supervised. The heart of the health district system is the health centre (*Centre de santé intégré*, CSI), which serves several villages and provides outpatient and inpatient care, including delivery. Health centres are managed by a nurse and overseen by a community-based management committee (COGES) made up of members elected by the villagers. Each COGES is responsible for managing, on a volunteer basis, cost recovery (CR) revenues generated under the Bamako Initiative (BI). The CSIs have two systems for processing patients: the BI system, and free services for the NGO targets. The first level of referral is the district hospital (where payment continues for all but services are free for referred children and pregnant women), which provides diagnostic services, outpatient and inpatient curative care, and caesarean sections and serves all the district's population. Cases that cannot be managed at the district hospital are referred to regional or national hospitals (where, again, payment continues and services are free for referred children and pregnant women). The NGO reimburses the transportation (to the district) and health services (to hospitals) costs for referrals of children and pregnant women carried out by the health district's ambulance.

Before the NGO's intervention was inaugurated, a community awareness and information campaign was undertaken in the villages, with the help of administrative and traditional authorities and local leaders. The intervention was not limited to fees abolition. The NGO wished at the same time to improve health care quality and availability. All required drugs and medical supplies were provided by the NGO on a monthly basis. The NGO's intervention thus ran in parallel with the BI cost recovery system, which continued to operate for patients other than children under five and pregnant women. The COGES managers ordered supplies from the central store. Thus, the CSIs had two pharmacies, one for free drugs provided by the NGO and another for those that were not free. To compensate for financial losses to the CR system related to the abolition of user fees, the NGO provided monthly operating grants to all health centres in both districts (average of 110 US$), and a monthly bonus (45 US$) to nurses, in addition to their governmental salary (140 US$), for extra clinical and administrative workloads. Clinical and administrative training was provided to them on-site. In sum, the NGO's intervention was not limited to abolishing payment, but consisted of a series of measures for improving quality of care (supply side: drugs, training, supervision, grants, referral, medical evacuation) and for implementing the abolition (demand side: fees abolition, information). While quality of care (notably access to drugs) is important for improving access to services, the rest of this article will focus essentially on an analysis of the implementation of what was an innovation for Niger and Francophone West Africa – the abolition of payment for all services for children under five years and pregnant women – from the specific perspective of the actors, using an anthropological approach.

## Methods

We used a mixed-methods approach, both qualitative and quantitative, to increase the internal validity through data triangulation. Three different data collection methods were used in April 2007.

### i) Individual in-depth interviews and focus groups

We questioned three categories of actors, conceived conceptually around the triangle of the decision [39] to abolish fees for certain services. These were the legitimizers (who formalize decisions), the actants (those who operationalize those decisions) and the beneficiaries (those who benefit from the decisions). In-depth (n = 85) and focus group (n = 8; 168 persons in total) interviews were conducted using an unstructured guide. The purpose of the interviews was to elicit the emic view of the impact of user fees abolition, the implementation process and the measure's sustainability. Interviews in the national language were conducted by a research assistant (physician anthropologist) from Niger and translated for the two investigators. Notes were taken systematically of all interviews to facilitate the analysis [40]. Because of time and resource constraints for this evaluation, the majority of the interviews were carried out in 12 of the two districts' 43 CSIs (30%), i.e., six per district. These CSIs were selected using criteria that provided contrasting cases [41] in terms of internal organization, quality of care, and utilization of services by the public. Several interviews at the levels of the Ministry of Public Health and funding agencies supplemented the information obtained from the peripheral regions. Table 2 presents the number of interviews by category of actor.

**Table 2:** Categories of actors and total number of interviews

		Individual during in-depth interviews			Individual during focus groups		
	Example	M	F	Total	M	F	Total

Legitimizers	Local officials, head of the Ministry of Health	15	0	15	4	5	9

Actants	Nurses, members of health committees, NGO staff, local manager	28	9	37	15	64	79

Recipients	Users and non-users	9	24	33	34	46	80

**TOTAL**				**85**			**168**

### ii) Participant observation

We carried out participant observation during the evaluation in these 12 CSIs, taking notes on the situations observed during periods of one to four hours. We attended curative consultations, preventive activities (immunization, growth monitoring, etc.), distribution of drugs, etc., in an effort to understand the dynamics of the interactions among the actors implementing this new intervention – patients, health care providers, and community authorities.

### iii) Questionnaires

During the evaluation, we invited all head nurses from the CSIs of both districts and members of the two district health teams (n = 57) to answer a self-administered structured questionnaire (adapted from [32]) to obtain their perceptions of the impact of user fees abolition, the implementation process. and the measure's sustainability. The majority of the questions used Likert-scaled attitude indicators, in addition to a few open-ended questions. The questionnaire was returned by 51 health staff (90%); among them 82% were male, 73% were between 31 and 50 years old, 95% were nurses and 5% physicians. On average, they had obtained their medical diplomas nine years before and had been in the districts for six years.

Data from the health staff questionnaire were analyzed using Excel^©^. The qualitative findings were analyzed using a thematic approach [41]. The preliminary results were presented to the relevant stakeholders in April 2007 in Niger, and the report of findings was submitted to the NGO and the chief medical officer of the health districts for comment in June 2007. The evaluation was conducted with the agreement and collaboration of the authorities in both health districts.

## Results

The following sections present the results of the analysis of this intervention, starting with the perception of its relevance and feasibility, then the implementation process, and finally the intervention's unintended outcomes.

### Perceptions about the judiciousness of user fees abolition

Opinions on the judiciousness of the fees abolition varied widely depending on the actors and can be summarized in three categories.

#### i) For the abolition

The group made up mostly of the populations and the target groups (beneficiaries) unanimously considered abolition to be justified. The abolition of fees produced peace of mind and encouraged people to attend health centres. In fact, some told us it was common for people to pawn their fields to obtain the means to pay for health care. Most persons we questioned did not seem to perceive any injustice in some people paying and others not. Children and women were seen as being vulnerable. Easing the financial burden benefited the entire household, which everyone considered to be positive.

### ii) Yes, but..

Health officials and local government decision-makers (legitimizers) were in favour of abolition because, in their view, it led to increased utilization of services. However, they worried that the State was relatively incapable of sustaining such a measure. They noted the difficulty of embedding the abolition of fees into the CR system, which itself had required a long time to organize and to get people used to paying. "The NGO's intervention is a wound that has already done damage," said one administrator. Thus we were told, in a phrase that was surprising coming from a Sahelian, that it was better "to teach people to fish than to give them fish." Their proposal therefore was that everyone should participate financially. Otherwise, they would prefer to see abolition introduced gradually, not suddenly.

#### iii) Against the abolition

Among health workers, opposition to free services was more widespread, although not total. They would have preferred to see drugs made available at lower prices and more accessible, rather than abolishing payment altogether. They asserted that abolition had provoked unwelcome behaviours among patients: lack of respect and of appreciation for services rendered, poor maintenance of health booklets, etc. They believed even a symbolic payment would be preferable to "disturbing a well established system that works well enough." The population's financial participation was still seen as a way of instilling a sense of ownership and respect for services. However, health workers were not unanimous in their opinion, since the statement "if patients don't pay for services, they don't value them," did not receive a majority of votes – 33% agreeing completely and 20% not at all (Table 3). Finally, other health care workers opposed free services because they said these had increased their workload.

**Table 3:** Health staff perceptions (in %)

**OUTCOME: Abolition has...**	**Agree completely**	**Agree moderately**	**Agree in part**	**Do not agree at all**
...improved service utilization for target populations.	94.1	5.9		

...had positive impacts on how I treat patients.	64	30	2	4

...strengthened my professional experience.	43.8	20.8	16.7	18.7

...allowed me to look after patients better.	63.8	23.4	6.4	6.4

...allowed me to treat more people than usual.	100			

...allowed me to help those who are most vulnerable.	94	6		

...reduced the time spent with each patient.	31.1	26.7	15.6	26.6

...increased my workload in terms of number of patients.	96	2	0	2

...increased my administrative workload.	83.7	8.2	4.1	4

...prevented me from seeing all patients who arrive.	12.8	14.9	17	55.3

...complicated my relationship with patients.	12.2	18.4	10.2	59.2

...obliged me to negotiate with patients who are not sick but want drugs.	63.3	10.2	10.2	16.3

...increased the number of patients who try to abuse the system.	64.7	13.7	9.8	11.8

...made me feel exploited.	31.3	16.7	12.5	39.5

...caused me to burn out professionally.	30	14	20	36

...made me frustrated.	6	12	16	66

...placed me under pressure from patients to give them more drugs.	28.6	24.5	12.2	34.7

...caused me to lose some of my personal income.	4	0	12	84

If patients don't pay for services, they don't value them.	32.7	24.5	22.4	20.4

The population confuses free services with the distribution of free drugs.	65.3	14.3	12.2	8.2

As a health professional, I felt personally affected by the implementation of free services.	83.7	16.3	0	0

**IMPLEMENTATION PROCESS**				

I was well informed of the procedures for the implementation of the free services.	77.6	12.2	0	10.2

Providing free services was more a decision of the funding agencies than of the government.	68	6	6	20

The strategies used to inform villagers and members of the community about the free services were well organized.	45.1	15.7	13.7	25.5

I was consulted for my opinion on how to implement the free services.	32	16	6	46

I think sufficient quantities of drugs have been provided to support the free services.	33.3	23.5	19.6	23.6

I think the drugs supplied to support the free services arrive on time.	28	40	22	10

The provision of free services would not have worked without financial measures to compensate for cost recovery.	46.9	18.4	6.1	28.6

The provision of free services could not have worked properly without bonuses for the personnel.	36.6	24.5	8.2	30.7

**SUSTAINABILITY**				

After the NGO leaves, the government will be able to sustain the system of free services.	29.8	12.8	27.7	29.7

I am confident that the government will be able to reimburse free services in the three months following the termination of the NGO's project.	12.5	22.9	22.9	41.7

I am confident that the government will be able to ensure the availability of enough drugs after the termination of the NGO's project.	8.3	18.8	18.8	54.1

### Perceptions about the sustainability of user fees abolition

In terms of sustainability, the general view that emerged was of abolition as a temporary phenomenon. The intervention was seen as an experiment or a project. One nurse said, "I have the impression that Niger has become a laboratory for testing all the systems, a guinea pig country." This view refers back to the experiment conducted in Niger that is often cited to justify CR policies [43]. In the popular imagination (after decades of experience with development projects), a project does not last; it is short-lived. One respondent from high up in the administration considered this project to be "assistance". One health worker at a health post, where free services are not yet offered as they are in the CSI, believed the State could not do it. According to him, this abolition was tied to a project. "You've seen it, it's foreigners who have come and given these free services," he told villagers, in justifying the fact that he continued to charge for services. There was also widespread concern about the State's capacity to carry forward this abolition introduced by an NGO. "Once HELP is over, things become risky," one nurse said. Nearly 30% of health workers thought the State could not keep it up. More than 40% believed the State would not be able to reimburse free services. Likewise, more than 50% believed the State will not be able to provide sufficient quantities of drugs once the NGO and its supply system come to an end (Table 3). Between scepticism and lack of confidence, the actors seemed to consider that a State-managed abolition of fees is destined for failure. The State's move to extend free services to the entire country was perceived as political. Among those we encountered, the political was often equated with deceitfulness. Others went so far as to propose a substitution: "We might just as well have HELP do it, because the State can't," said one village treasurer.

### Strengths of the implementation process

#### i) A top-down information and community awareness phase

Local authorities largely participated in the dissemination of information on the abolition. The NGO relied on both traditional and administrative channels of information to explain clearly their objectives and the target groups for their activities. The usual procedures were followed with utmost respect to involve local leaders and decision-makers in the information phase. Admittedly, they were involved very little, or even not at all, in the decisions and the choice of intervention modalities, which some people, particularly the nurses, deplored. Nevertheless, their involvement in the top-down strategy definitely helped in disseminating information. Also, the NGO was able to organize space where they could provide local partners with regular updates on their activities; for some officials, this brought real added value to this NGO, as compared to others about which, according to one prefect, very little was known.

### ii) Incentive measures

The NGO perfectly anticipated the reluctance of those involved in the implementation, by organizing incentive measures substantial enough to limit abuses – although not all of them, as we shall see later. The financial data show that the operating grants to the CSIs were sufficient to avoid any de-capitalization that would have endangered the COGES' sustainability. According to official data, the rate of cost recovery remained above 100%. Everyone involved appreciated that the salaries of the community fees collectors and the COGES treasurers remained more or less at the same levels as before. In some CSIs in Mayahi and all CSIs in Tera, this grant even allowed these persons to earn more.

#### iii) Drug supplies

Overall, everyone agreed the supply of drugs was well organized. This was all the more appreciated because the State system does not work well. Admittedly, there were some shortages and occasional problems in adjusting the supply with unrealistic demands by nurses at the beginning of the intervention. The cost recovery drugs were often stashed in cupboards while those from HELP were kept at hand for the many consultations. In addition, new drugs appeared in the prescriptions of health workers who understood them: ocytocics, magnesium sulfate and, of course, sulfadoxine-pyrimethamine, which replaced chloroquine for pregnant women, as well as the other antimalarials that were replaced. The NGO trained all health workers in the rational use of essential generic drugs and in new treatment protocols (such as stopping the use of chloroquine).

#### iv) Medical evacuation system

Everyone acknowledged HELP's very significant role in medical evacuation and in rapid and free response to emergencies. Evacuation was being done quickly, whereas before, under the cost recovery system, patients had to return home to find the money required.

### Weaknesses of the implementation process

Beyond these strengths, certain limitations were also raised. We focus here on the most important, in terms of lessons applicable to other interventions.

#### i) The information and community awareness phase

Because the process relied primarily on local authorities and leaders, others such as community officials, COGESs, and street level workers were sometimes left out. Thus, there were breakdowns in transmitting information to villagers. The NGO left this responsibility to village chiefs and sometimes to CSI nurse managers, who did not always carry it out well. One woman said, "When they introduced free services, they didn't call us together to tell us about it; I learned about it from women who came for prenatal visits." Some people only found out at the CSI, when it came time to pay. Also, the quality of information was sometimes inadequate, and the announcement of the free services was sometimes incomplete or poorly explained to the public. For instance, while some women we encountered knew what the target groups were, they did not always know what services were covered by the abolition.

#### ii) Incentive measures

Despite the positive aspects of the incentive measures described earlier, two problems nevertheless arose. The first concerned the fact that some CSIs had guards who were paid from cost recoveries. Because these salaries were not covered by the HELP grant, some CSIs continued to charge each patient 25–50 F CFA (0.5–0.10 US$) to pay for the guard. The second problem had to do with the *per diem *spiral, a perverse effect that is well known in development projects [42,43]. To ensure the CSI managers would accept the intervention, and on the pretext that, according to them, this would increase their workload, the NGO agreed to give them an "incentive" – a monthly bonus of 20000 F CFA (45 US$). However, the State will not be able to assume this cost and the workload excuse does not appear to be justified at the time of this evaluation. The CSI managers were asked to share this bonus with the other workers, but this was not done everywhere.

#### iii) Lack of COGES involvement in the system

The COGESs were not always kept informed and rarely participated in the implementation. Thus, the CSI managers are the masters of the system. A glaring example of this low level of involvement was the spontaneous reaction of the members of one district team to our desire to assemble the COGES presidents for a group discussion on fees abolition: "But the presidents don't know anything!" While it may be that most district COGESs do not function well, they were certainly not strengthened by the intervention and, in some cases, may even have been destabilized.

#### iv) Introduction of parallel operating systems

One of the major weaknesses of the project was the creation of two parallel operating systems, rather than integrating this intervention into the existing system using a systematic approach to support the district health team. Unfortunately, as one chief physician told us, "they have nothing in common." Fees abolition became a vertical program, like vaccination. Nurses had to manage different groups of registered patients, some in the CR system, some in the HELP system. We could even say there were multiple functions, having observed that CSIs already had the experience of free services provided by other NGOs (Islamic Relief, World Vision) or in other programs (tuberculosis, leprosy, etc.). Concretely, as one nurse said, in the CSIs there was "a BI table and a free services table," where drugs were provided, but "it's very difficult for one worker to be applying two different management systems." For many people, this clear distinction was justified for accounting transparency and for the NGO's visibility. Also, as one nurse said, "with HELP, the BI was completely turned upside down." A recurrent complaint was the cumbersome bureaucratic necessity of filling out administrative documents in duplicate so the funding agency could have the specific data it required. Moreover, district teams were not really involved in the NGO's activities. They did not receive copies of the drug stocks received, participate in their distribution, or carry out coordinated supervision.

#### v) Lack of action to reinforce the supply side

The abolition of fees undoubtedly acted upon demand by reducing the financial burden. Supplying drugs to the CSIs allowed the supply to respond, but only in part, to the demand. Also, the NGO had not done enough so that the supply could respond better to increases in demand. Sending in an intern to supplement human resources was a late decision, but not a sustainable solution. There was not enough institutional support in terms of equipping and reinforcing the health system, despite the supplies of basic operating materials.

#### vi) Lack of follow-up in the medical evacuation system

Setting up the medical evacuation system was a major accomplishment of this project. However, there was not adequate follow-up to ensure that the women for whom the NGO reimbursed services at the referral centres actually received these services at no charge. Some women complained, "Before, we had to pay 20000 F CFA for gas; now it's free, but when we get to the Regional Hospital, we have to pay." Thus, there was the risk that women's hopes, raised by the organization of medical evacuation services and free caesareans, would collapse because of poor follow-up and management inconsistency between HELP on one side and the State on the other.

#### vii) Coordination of the levels of free service in the health pyramid

Similarly to the problems in the evacuation system, the modalities for fees abolition had not really been coordinated between the various levels of the health pyramid. Apparently the funding agency requested that the NGO not intervene in the hospitals and health posts. Thus, services are free in CSIs for women and for children under five, but if they are evacuated or go on their own to a health post at a higher level in the hierarchy, or if they come from a lower level in the pyramid, they have to pay.

### Actor's perceptions of outcomes

Table 3 presents the results of the questionnaire administered to the health personnel. With respect to patients, the personnel clearly saw a positive impact of fees abolition on service utilization; 94% agreed completely with the proposition of an increase stimulated by the abolition of fees. All (100%) believed this had resulted in more people being treated than before. Moreover, they said the abolition had helped the more vulnerable. They also believed the abolition had some negative impacts on patients. The same proportion (two-thirds) thought that: i) abolition had increased the number of patients who try to abuse the system; ii) people confused abolition with free distribution of drugs; and iii) more people now tried to obtain drugs when they were not sick. For example, one nurse said, "Some women arrive with the name of the drug they want." Regarding impacts on their professional practice, more than 80% of the workers stated that they felt personally affected by the implementation of the abolition. 64% stated that this had positive impacts on how they treated patients (improved quality). However, a majority of staff complained of the added administrative and clinical workload. But based on our observations in the field, we calculated that a nurse spent an average of eight minutes with each patient. This corresponded to about 4.1 hours of curative work per day in Mahayi and 2.6 hours in Tera. In Mayahi, the maximum number of consultations per day after fees abolition was 31, in the fourth quarter of 2006, and 20 in Tera, in the third quarter of 2006. Also, a majority (55%) did not think the abolition prevented them from treating everyone who came. In summary, they told us there was more work but no reduction in quality, nor selection of patients treated. No consensus emerged on perceptions of the extent of personal impacts, on operations, on professional burn-out, or on pressures experienced. Table 4 (based on this questionnaire and qualitative data) summarizes workers' perceptions of the intervention's outcomes, as well as what we heard from women and members of the management committees.

**Table 4:** Summary of the actors' perceptions of the impacts of fees abolition

	Women	Health workers	Members of management committees
Positive impacts	Promotes medical evacuation Reduced costs of treatment	Increased number of visits Earlier visits Promotes medical evacuation Emergence of new drugs Helpful arrival of NGO interns	Brings comfort to the target populations

Negative impacts	Abolition does not eliminate all costs Multiple-dose medications require repeat visits to the CSI Health workers' sometimes scornful attitude, assuming that women are pretending to be ill to obtain drugs	Patients in the target groups becoming more demanding	Nurses monopolizing the management of inputs Management committees pushed aside

Workers' negative perceptions of women's supposedly improper behaviours have consequences, women said, on how they are received by the nurses. One patient explained,

"Even though it's free, before it started, and now, it's not the same. Before, when you came, they gave you a tube of ointment, but now, you bring your child in the morning and again in the evening for them to put the medicine in his eyes."

Another woman made it clear that abolition did not always mean things were free, since, "Before, we gave 800 francs and today we give 200 francs, it's as if they have lowered the price. So we can say '*Alhamdu lilahi*' (Thanks be to God)." Members of the COGES also were not pleased that drugs related to the abolition were managed by the nurses and no longer by the management committees created by the BI.

### Some unintended/undesirable outcomes

Our aim in this section is to describe some unexpected outcomes provoked by the abolition of fees implemented during this NGO intervention – a type of analysis rarely undertaken, according to certain experts in evaluation [44]. The social actors adapted to this financial innovation in ways designed to minimize disadvantages to themselves.

#### i) From the population's perspective: medicines associated with the distribution of food aid

Two years before this health intervention, the NGO had begun its action in Niger by distributing food supplies during the food crisis in 2005. Thus, the abolition of user fees, and the abundance of new consultants and drugs to cope with it, was sometimes interpreted by the population as a distribution of medicine. As with food aid, where the organizers are very aware of pilferage, "there was lots of wastage" in the first weeks of the intervention, one nurse told us. Thus, not knowing whether this windfall would continue, or to make sure they would have medicines for when their children actually became sick, some patients apparently came to the centres to build up a reserve of medications; "there's a big rush on, because it won't last," said a nurse. Thus, according to the nurses, there was a phenomenon of stockpiling.

#### ii) From the perspective of the healthcare workers: strategies for recuperating the shortfall

Health care workers have always organized parallel systems to boost their incomes. These parallel practices were integrated into a system where people paid for everything. Thus, the act of abolishing some fees and informing the population of that fact made these strategies more complicated (but not impossible) to carry out. Nevertheless, the health care workers managed to adapt perfectly well to the new situation. All of them insisted that the abolition of fees greatly increased their workload "to the point of irritation" and reduced the time available for each patient – a claim that was not borne out by our observations. These statements are somewhat exaggerated; the most motivated workers managed to better organize the distribution of tasks and the roles of the health personnel. Actually, the strategy behind these statements is to pressure the NGO to recognize that they are "overwhelmed" and consequently to increase the bonuses they receive for working in the free system. Some nurses redirect the free drugs from the NGO into the fee-for-service system that continues for other categories of the population who are not beneficiaries of the project. Creating artificial stock shortages of goods supplied for free by the NGO is another way of getting around the NGO's rules. By forgetting to replenish the stocks of health booklets, nurses will create a shortage that will allow them to purchase the same booklets manufactured in neighbouring Nigeria, which they then resell to patients privately at a profit. Others are even more creative. On the pretext that the women do not take proper care of the health booklets, some nurses have "required that the booklets be plastified," said one manager, for the same price at which they used to be sold. Other nurses write their prescriptions on a piece of paper that they staple to the booklet, then charge 25 F(0.05 US$) for the staple. We were thus not surprised to see a nurse open, inadvertently, a drawer filled with coins in his office. The other solution is simply to charge for certain services that are free. One woman told us, "I paid 1,000 F (2 US$) for a delivery a few days ago." Another woman recounts that she paid for her first prenatal consultation; but the health workers had chided her for coming in, because they took advantage of the rural inhabitants' lack of information to charge them when they came into town for services, so they said to her, "Hey, city-dweller, why did you come today? Today is for the peasants, they pay cash, so you'll have to pay, too." Some CSIs continue to charge each patient 50 F or 25 F to pay, we were told, the salary of a guard. Thus, one woman reported having been charged several fees: "I didn't have to pay for the *awo *[antenatal care], but I had to pay for the booklet. So, I paid 100 francs for the booklet, 100 francs to have it plastified, and 25 francs for the guard." When we asked women who were waiting in line in front of a CSI why the services had become free, the response suggested to us that it was not always so: "It's because you are here today." We were also told that the abolition of fees "created problems of misunderstanding between the health workers and the population."

#### iii) The provider-patient relationship: lack of understanding

The abolition of user fees had several impacts on medical practice and particularly on the interaction between provider and patient. Many patients consider that the medicines supplied in the free system are, in effect, owed to them by the NGO and made available through the CSIs, and that health workers are only intermediaries whose role is to distribute them. This lack of understanding about the abolition of user fees has led users to develop strategies for hoarding medicines. Thus, the majority of nurses (63%) completely agreed with the statement that abolition required them to deal with patients who were not sick and wanted to abuse the free system. According to the nurses, patients have adapted their strategies for acquiring medicines. Some pretend to be sick, and others, who arrive with a healthy child, listen to the description of the symptoms of the mother ahead of them in line and say the same things that will help them get the medicines they want. Since many nurses do not systematically take vital signs (none that we observed did so) and provide care based only on reported symptoms, the likelihood that mothers will be given medicines is quite strong. Some people go from one CSI to another. The massive arrival of cough syrup was perceived by mothers as a great opportunity because they associate it with vitamins. Thus, as one nurse reported, "we were obligated to give the cough syrups to the mothers." Moreover, health workers say patients have become more demanding and insist on receiving the treatment of their choice. They arrive late at night, even for a mild cold. We did not observe any such behaviours during our observations in 12 CSIs and can only repeat here what was reported by the nurses.

However, patients are not happy, either, with how they are treated. Complaints about health workers are frequent. For example, they complain that workers ration the medicines. In addition, they find that the workers are scornful toward them, treating them as though they are pretending to be sick and only come to the CSI to get medicines.

## Discussion

### Methodological limitations

It is clearly not our intention to generalize our results to all interventions in Africa aimed at abolishing fees. Nevertheless, this intervention, while limited to two districts, involves one million inhabitants and more than 40 health centres. In addition, this is an NGO intervention (NGO bias), using a relatively vertical process, and not that of the State. As the government of Niger decided in 2007 to generalize the abolition of fees for prenatal care and children under five years (but not for deliveries), it will be interesting to document the alternative implementation process, the funding modalities (output-based payment rather than supplying inputs), and the authoritarian role of the State (vs. NGO) concerning some health workers' practices. Still, this study is important to document the various actors' perceptions and reactions to this innovation, the abolition of fees. Even if the districts are nearly 1000 km apart, most of the results were similar in both, with very few exceptions (more informal payments, a little more integration in the district health team's activities, a more advanced community information phase), which gives them a certain internal validity. The stakeholders validated and discussed the results at a dissemination workshop in 2007.

### Practical strategies for managing fee removal

This is one of the first studies to document the process of abolishing fees by an NGO for children under five years and pregnant women in a context where cost recovery, under BI principles, was still operating and ongoing among those populations not targeted for the intervention. The results show that the implementation of the intervention, of which fees abolition was the core – but not the only – instrument, unfolded relatively well, even if there are still many elements to be improved. Removing one of the most serious barriers to care for women and children is a praiseworthy initiative. Everyone, in fact, acknowledges that in the African context, and especially with respect to women's rights [45,46], removing this barrier can have significant impacts on service utilization [29,47-49]. The cost of health care is a major source of anxiety among women, leading them to postpone care and bringing the poor deeper into debt [50]. Clearly, the effectiveness of the project is not due solely to the abolition of fees, since the NGO also acted on improving the quality of care which, in the present case, essentially meant ensuring the availability of drugs, paying bonuses to COGESs and nurses, and organizing a medical evaluation system. The existence of these inputs is indispensable to this type of intervention, as is clearly shown in the experiences of Ghana, Zambia and Uganda [17,26,30,51].

In Burkina Faso, where the Bamako Initiative is at the heart of the health system, integration of the recent policy of subsidizing 80% of the cost of deliveries and caesareans within the cost recovery system was also well operationalized, even though the impacts on utilization are heterogeneous [52]. Maintaining the incentives for health workers, the integration into the existing system of drug distribution, and output-based payment managed by the COGESs enabled the policy to be organized without too much resistance, despite some trial and error in the strategy's content. Conversely, in Senegal, the lower-level facilities never received any financial reimbursement for lost fees from the start of free deliveries. Researchers also report shortfalls and delays in the arrival of delivery kits. One consequence was that health facilities at the district level lost 4–15% of user fee revenue and coped by "increasing tariffs for others services" [19] and looking for other alternatives.

Obviously, in Niger, improving the quality of care by these inputs alone is necessary, but not sufficient. There is still much to be done, especially in obstetric services, whose quality has been very limited in Niger for a long time [53,54]. Another very positive aspect of this intervention is the organization of a referral-evacuation system. We know that, to act on maternal mortality, reducing financial barriers to access is not enough, and promoting the evacuation of women is primordial [3,47]. In Niger, this system functions rather poorly and the two most significant barriers are "the hurdles of finding transport and of paying for it" [55]. The intervention reinforced the national policy, decided in 2005, of abolishing user fees for caesareans because these districts do not always have the means to operate ambulances. Nevertheless, there are limitations related to the fact that we uncovered cases of some women who were evacuated to the regional hospital at no charge, but then apparently had to pay certain amounts, which is obviously not the NGO's fault. This situation was also found in Senegal, where products that were supposed to have been part of the free delivery kits were paid for by the persons we interviewed [19]. Beyond the debates on informal payments, this illustrates once again the importance of considering the health care system in its entirety when taking action. Finally, if we go back to the seven strategies for managing fee removal proposed by the experts [30] based on past experiences, many were respected in this intervention:

• good communications with key actors;

• retaining local management of revenues from services still being charged;

• a good public information campaign;

• an adequate supply of inputs (drugs, personnel).

### Unintended consequences

Xu et al., in evaluating the Uganda experience, stated that the abolition of fees can have "unintended consequences" [23]. Our qualitative approach, based on an anthropological type of survey procedure, allowed us to highlight some of these unintended consequences. While there must be other types, we focused essentially on the interactions among actors, and particularly among nurses and patients. In no way could it be said that any of the behaviours observed in this study were created by the project. They existed before and will certainly continue afterward but the project could have exacerbated them. In Niger, "the patient-nurse relation is characterized by authority and passivity" [55], as has been shown by many anthropological studies [53,54]. The practices we describe show essentially that a change (the abolition) allowed the social actors to adapt (coping strategies) and transform their behaviours in line with the new situation in such a way as to preserve the status quo. We have an empirical demonstration of the actors' margin of maneuver, to use Strauss' expression, exercised by both the nurses and the women. In South Africa, nurses also claimed that some patients abused the new system [32]. In Ghana, unlike in Niger, it appears informal payments were reduced after the abolition in one region, while data on this are mixed in another [17]. Perhaps the Nigerien context is particular? An analysis of food distribution during the 2005 food crisis in Niger revealed the same types of coping strategies when the "development income" arrived [56]. Women "rented" themselves out as wives to men when food assistance was aimed at families and not at unmarried men. Malnourished children were considered "lucky babies" in villages because they opened the door to multiple forms of aid; some women "borrowed" such children or tried to "provoke diarrhea to make the child thinner", in order to receive aid. On their side, some unscrupulous workers sold the bracelets that gave the bearer the right to food aid. It would be useful in the future to study whether the fact that user fees abolition is now organized at the national level by the State and not by an NGO could have positive impacts on such practices; the issue will be that of the State's authority.

### Street level workers and implementation

Previous studies on the abolition of fees showed that the role of health workers as street level workers is fundamental in successfully operationalizing this type of reform, and that understanding their perspective as implementers is essential [17,32], as this study of Niger also shows. In South Africa, health workers had a negative view of free care implementation [32]; in Ghana and in Zambia, they worried about sustainability [26,57]; and in Uganda, they did not appear to be any more satisfied with their work after the abolition of fees [21]. This study in Niger shows that the workers were satisfied with how the intervention had unfolded and, as in other countries, they considered it to be a very good policy for improving access to services for the poor. If they seemed more satisfied than others elsewhere, it may be because their working conditions actually improved with the arrival of the NGO. Finally, CSIs had drugs when the national drug supply system (ONPPC) was operating chaotically, and the financial incentives were much appreciated. The same strategy was tried successfully in Zambia, where the districts all received financial compensation that was apparently sufficient [26] and in Ghana, where 88% of the health workers reported an increase in their monthly salaries, which was unconnected to the user fees abolition [57]. In all these experiences, nurses also worried a great deal about heavier workloads [17,32,57], even when there was very little evidence of such increases. In this experience in Niger, contrary to the situation in South Africa [32] and the assertion of the Nigerien nurses, the hypothesis that there had been a workload increase due to the abolition was questionable (except on market days). Based on our observations done in April 2007, there was still quite a large margin for maneuver before the nurses would be overloaded. That being said, the observation period was brief and it will be important to ensure that any increase in visits remains workable for the nurses and midwives. Otherwise, an increase in human resources will become unavoidable, although not insurmountable, as was demonstrated in Zambia, which recruited more than 1300 new staff (mainly nurses) [26]. Increasing the number of skilled attendants remains a priority in Africa, if maternal mortality is to be reduced [3,58].

### Context sensitivity, Bamako Initiative and sustainability

The results of this study confirm the lessons drawn from other African countries about the importance of the process. However, the context is different in Francophone West Africa because the BI (a community-based cost-recovery scheme) has been in place a long time. Is the process described in this paper sustainable? Conceptually, two types of specific events favour sustainability: i) stabilization of the organizational resources allocated to programs; and ii) risk-taking by organizations in favour of programs and integration of activities [59,60]. During its intervention, funds provided by the NGO to compensate for the loss of revenues from user fees allowed the CSIs to maintain their levels of cost-recovery (data not shown). Funds accumulated since the country's inauguration of the BI were thus not touched (300 million F CFA [650000 US$] in 2002, according to the Ministry of Health [61]). In Zambia, funds allocated by the government to compensate for lost user fees increased district revenues by 36% on average [26]. In 2007, the Government of Niger decided to generalize abolition of fees for children under five (but not for assisted delivery) at the national level and voted in the necessary budgetary appropriations (in addition to donor funding). This national application remains to be evaluated. However, the government had not first well prepared the implementation phase, nor had the NGO taken the organizational risk of integrating the abolition into the CR system established by the BI. The local management committees were not sufficiently involved, as they were in Zambia [26]. Yet these committees had spent years setting up the system and informing the local populations. They also continue to manage the system for those categories of patients that still must pay. Thus, in defining the modalities for organizing the abolition, the national context must be taken into account more carefully – even more so, since those who implemented the BI are the most sceptical about the judiciousness of abolishing fees. The success and the continuation of the abolition of fees, if the quality of services and the funding are guaranteed, may help to restore the population's confidence in the State and its health care system [62]. Yet, in Niger, most of the people we encountered had no confidence in the State. Corruption in many public sectors in Niger, including in health care [53,54], is well documented [63]. Still, "the population looks up to them [nurses] as representatives of the 'State"' [55]. The abolition of fees may be one vehicle for restoring this confidence.

## Conclusion

Abolishing user fees in the context of the BI in West Africa is not an easy decision. Many Francophone West African states whose health care systems are based on the BI are considering it. As of 2007, only Niger had applied this policy nationwide for children under five, after the NGO intervention in two districts in 2006–2007. This article describes the fundamental importance of the implementation process in the success of such an initiative in the BI context. In a system where some patients pay and others do not, the interaction between the two parts of the system needs to be carefully considered and organized in line with the local reality.

After this study, the NGO reacted perfectly and adjusted its intervention to integrate it better into the national system by supporting the State in implementing this new national policy. In fact, one lesson learned from this study is that it is important, when introducing a reform, that planners involve the health system as a whole, defining service delivery modalities at all levels in the system. This was the solution chosen by Niger in extending abolition for children under five to the national level. The State decided to insert itself into the system set up under the Bamako Initiative. It must reimburse health centres and management committees on the basis of a standard amount per consultation, since the centres need to go through the usual channels to obtain their drug stocks. The NGO continues to support the two districts, focusing its activities on quality improvement and medical evacuations. The impact of this national policy on children remains to be analyzed, since the State is still debating whether to abolish payment for deliveries in the country. In addition, if the worst-off are really to benefit, it is essential to ensure, at the same time, that quality is improved and that mechanisms are in place to prevent abuses. While current knowledge about the impacts of fees abolition seems clear enough, there is still much work to be done to document processes in West Africa. This work is important because ultimately, once the decision is taken, it is the processes that determine success.

## Competing interests

The authors were recruited as consultants by the NGO. Neither the NGO nor its funding agency had any influence on the results presented in this article.

## Authors' contributions

VR and AD wrote the evaluation protocol, collected and analyzed the data and wrote the final report. VR wrote the first draft of the article. All authors read and approved the final manuscript.

## Pre-publication history

The pre-publication history for this paper can be accessed here:


